# PTEN-induced putative kinase 1 regulates mitochondrial quality control and is essential for the maturation of human induced pluripotent stem cell-derived cardiomyocytes

**DOI:** 10.1016/j.gendis.2022.08.023

**Published:** 2022-09-10

**Authors:** Huiwen Liu, Yanting Sun, Hao Xu, Bin Tan, Qin Yi, Jie Tian, Jing Zhu

**Affiliations:** a Department of Pediatric Research Institute, Children's Hospital of Chongqing Medical University, Chongqing 400014, China; b Chongqing Key Laboratory of Pediatrics, Chongqing 400014, China; c Department of Clinical Laboratory, Children's Hospital of Chongqing Medical University, Chongqing 400014, China; d Department of Cardiovascular (Internal Medicine), Children's Hospital of Chongqing Medical University, Chongqing 400014, China

**Keywords:** hiPSC-CMs, hiPSCs, Maturation, Mitochondrial quality, PINK1

## Abstract

Human induced pluripotent stem cell-derived cardiomyocytes (hiPSC-CMs) have attracted attention in the field of regenerative medicine due to their potential ability to repair damaged hearts. However, the immature phenotype of these cells limits their clinical application. Cardiomyocyte maturation is accompanied by changes in mitochondrial quality. PTEN-induced putative kinase 1 (PINK1) has been linked to mitochondrial quality control. However, whether the changes in mitochondrial quality in hiPSC-CMs are associated with PINK1, and the impact of PINK1 on hiPSC-CMs development are not clear. In this study, we found that knockdown of PINK1 in hiPSC-CMs resulted in mitochondrial fragmentation and impaired mitochondrial functions such as mitophagy and mitochondrial biogenesis. PINK1 deletion also inhibited the maturation of hiPSC-CMs, reverting them to a naive structural and functional state. We found that restoring the mitochondrial structure did not completely rescue the mitochondrial dysfunction caused by PINK1 deletion, while activation of PINK1 kinase activity using kinetin promoted mitochondrial fusion, increased the mitochondrial membrane potential and ATP production, and maintained the development and maturation of hiPSC-CMs. In conclusion, PINK1 regulates the mitochondrial structure and function of hiPSC-CMs, and is essential for the maturation of hiPSC-CMs.

## Introduction

Human induced pluripotent stem cells (hiPSCs) have multidirectional differentiation potential and can rapidly differentiate into beating cardiomyocytes (CMs).^1^ However, human induced pluripotent stem cell-derived cardiomyocytes (hiPSC-CMs) are structurally and functionally immature,^2^ which prevents them from being widely used in the clinic.^3^ Studies have shown that the maturation of hiPSC-CMs is promoted by modulating energy metabolism, such as by the addition of fatty acids^4^ or hormones,^5^ but excessive nutrient accumulation can cause cytotoxicity.^6^

Most of the energy consumed by the body is provided by mitochondria, which play an important role in sensing and transforming available metabolic substrates.^7^ During cardiac development, mitochondria shift from a short rod-like shape depleted of mitochondrial cristae to an extended network-like shape enriched in mitochondrial cristae to provide sufficient ATP for cardiac contraction.^7^^,^^8^ This phenomenon suggests that the changes in mitochondrial structure and function are not only a response to CM maturation, but may also promote CM maturation. The mechanism by which mitochondria regulate CM maturation during development needs to be elucidated to identify strategies to promote the maturation of hiPSC-CMs and increase the application of these cells in clinical practice.

PTEN-induced putative kinase 1 (PINK1) is a serine/threonine kinase with important biological roles in the regulation of mitochondrial quality. One of the most widely studied processes involving PINK1 is PINK1-mediated mitophagy, through which damaged mitochondria are cleared.^9^^,^^10^ In addition, accumulating evidence indicates that PINK1 affects mitochondrial oxidative phosphorylation and energy supply by regulating mitochondrial biogenesis,^11^ fusion, fission^12^^,^^13^ and the electron respiratory transport chain complex.^14^ PINK1 is extensively expressed in the adult heart^15^ and is important for mediating mitochondrial renewal and the maintenance of a normal energy supply to regulate heart development.^16^^,^^17^ In addition, PINK1 has been shown to mediate the development of *Drosophila*.^18^ These findings suggest that the regulation of mitochondrial structure and function by PINK1 is essential for heart and organism development, but it is unclear whether PINK1 affects mitochondrial structure and function, and whether it regulates the maturation of hiPSC-CMs.

We hypothesized that PINK1 deficiency leads to defects in the mitochondrial structure and function, thereby inhibiting the maturation of hiPSC-CMs. We herein demonstrate that PINK1 deletion resulted in mitochondrial fragmentation in hiPSC-CMs. Furthermore, PINK1 deletion led to mitochondrial abnormalities, and significantly reduced the maturation of hiPSC-CMs, resulting in a shortened sarcomere length, a reduced cell size, a more rounded cell morphology, and abnormal cell function. Moreover, the induction of mitochondrial fusion rescued the mitochondrial fragmentation caused by PINK1 deficiency, but did not effectively improve mitochondrial function. In contrast, activating PINK1 kinase activity alleviated the impairment of hiPSC-CMs maturation caused by PINK1 deficiency. Thus, this study demonstrates that PINK1 is regulates the quality homeostasis of mitochondria and involved in hiPSC-CMs maturation.

## Materials and methods

### Culture and differentiation of hiPSCs

The hiPSCs used in this study were reprogrammed from human urine-derived renal epithelial cells (CELLAPY, PR China). The hiPSCs were inoculated in culture plates coated with matrix gel (Corning, USA) and were cultured in PGM1 medium (CELLAPY). Differentiation was induced when the cells reached 90% confluence. On day 0, 6 μM CHIR (Selleck, USA) was added to RPMI 1640 (Sigma, USA) with B27 supplement minus insulin (Thermo Fisher Scientific, USA), mixed well and added to the culture plate, and this medium was replaced with RPMI 1640 with B27 supplement minus insulin after 48 h of incubation. On day 3, 5 μM IWP2 (Selleck) was added to RPMI 1640 with B27 supplement minus insulin, mixed well and added to the culture plate, and this medium was replaced with RPMI 1640 with B27 supplement minus insulin after 48 h of incubation. On day 7, pulsating CMs were visible after the addition of RPMI 1640 with B27 supplement with insulin (Thermo Fisher Scientific), and the medium was changed every three days. On day 13, 4 mM lactic acid (Sigma) was added to glucose-free RPMI 1640 (Thermo Fisher Scientific) with B27 supplement with insulin, mixed well and added to the culture plate for the selection of CMs because CMs can use lactic acid as a metabolic substrate, while other cell types cannot.^19^ On day 16, the medium was replaced with RPMI 1640 with B27 supplement with insulin to rescue the CMs from starvation. On day 18, the CMs were replated at a ratio of 2:1 or 3:1 in culture plates coated with matrix gel, and the medium was changed after 24 h. The CMs were subsequently maintained in RPMI 1640 with B27 supplement with insulin.

#### siRNA transfection

The siRNA used in this study was human-derived siPINK1 produced by RIBOBIO. Cells were inoculated and grown to 60%−70% confluence, and the siRNA was diluted in OptiMEM (Thermo Fisher Scientific) according to the instructions for RNAiMAX (Thermo Fisher Scientific). The transfection reagent RNAiMAX was diluted in OptiMEM, and then the diluted siRNA and RNAiMAX were mixed at a ratio of 1:1, incubated for 5 min at room temperature, added to cells and incubated for 6 h. Then, the solution was changed, and the cells were incubated for 42 h, after which the next step of the experiment was performed. The concentration of siPINK1 used in this study was 100 nM. The sequences of siPINK1 are shown in Table S1.

### Drug treatment

M1, Mdivi-1 and kinetin were obtained from MCE. Predetermined concentration gradients were used to identify the optimal concentrations of the M1 (1 μM, 5 μM, 10 μM, 20 μM, and 50 μM), Mdivi-1 (5 μM, 10 μM, 25 μM, 50 μM, and 100 μM), and kinetin (1 μM, 5 μM, 10 μM, 20 μM, and 50 μM) treatments. We ultimately used 10 μM M1, 100 μM Mdivi-1, and 1 μM kinetin as the experimental concentrations. After 6 h of treatment with siPINK1, the medium was changed, the drugs were added individually, and the cells were incubated for 42 h for use in subsequent experiments.

### Immunofluorescence staining

Cells were washed with PBS before staining. The cells were fixed with 4% paraformaldehyde for 20 min and washed 3 times with PBS. Triton X-100 was added to permeabilize the cells for 10 min. After blocking with 5% bovine serum albumin (BSA) at room temperature for 30 min, tdiluted primary antibodies (Table S2) were added, and the cells were incubated at 37 °C for 2 h, after which they were washed 3 times with PBS. Diluted secondary antibodies (Table S2) were added, and the cells were incubated for 1 h at 37 °C, after which they were washed 3 times with PBS. Diluted Hoechst 33,342 (Beyotime, PR China) was added, and the cells were incubated at 37 °C for 30 min, after which they were washed 3 times with PBS. Anti-fluorescence quenching agent was used to seal the slides, and the slides were photographed using a 90I inverted fluorescence microscope (Nikon, Japan) or A1R confocal microscope (Nikon, Japan). Cell morphological indicators were statistically analyzed using ImageJ software. More than 30 cells were used for each experiment, and each experiment was repeated three times.

### MitoTracker and LysoTracker staining

Working solutions of MitoTracker Green (1 mM; Beyotime) and LysoTracker Red (1 mM; Beyotime) were prepared by diluting MitoTracker and LysoTracker to 0.2 μM and 50 nM, respectively, and prewarmed to 37 °C. The medium in the confocal dish was discarded, the cells were washed with PBS, 950 μL of MitoTracker working solution was added, and the cells were incubated for 15 min at 37 °C. The working solution in the confocal dish was discarded, the cells were washed 3 times with PBS, 1 mL of LysoTracker working solution was added, and the cells were incubated for 30 min at 37 °C and washed 3 times with PBS. After that, 1 mL of fresh medium was added, and images were captured using an A1R confocal microscope. For the mitophagy assay, cells were pretreated with 10 μM FCCP (Topscience, PR China) for 1 h before being stained. Mitochondrial morphology was statistically analyzed using ImageJ software.^20^^,^^21^ More than 30 cells were used for each experiment, and each experiment was repeated three times.

### Mitochondrial membrane potential (ΔΨm) measurement

The ΔΨm was determined using JC-1 dye (Beyotime). A total of 50 μL of JC-1 (200×) was added to 8 mL of ultrapure water, and then 2 mL of JC-1 staining buffer (5×) was added to make the JC-1 staining working solution. JC-1 working solution was added, and the cells were incubated for 20 min at 37 °C. After incubation, the cells were washed twice with JC-1 staining buffer (1×), 2 mL of fresh medium was added, and images were captured with A1R confocal microscope. The average fluorescence intensity of the JC-1 monomer (excitation wavelength of 514 nm) and JC-1 polymer (excitation wavelength of 585 nm) was measured with ImageJ software, and the 585/514 ratio was calculated.

### Measurement of the cellular ATP level

Intracellular ATP levels were measured using an ATP assay kit (Beyotime). Lysis buffer was added to the culture plates, and the cells were lysed on ice. After lysis, the supernatant was removed by centrifugation at 12,000 r/min for 5 min at 4 °C. A total of 100 μL of ATP assay working solution was added to each well of an opaque 96-well plate and incubated at room temperature for 5 min. Then, 20 μL of the sample to be tested or ATP standard solution was added. The relative light units values were determined using a Cytation 5 (Biotek, USA).

### Cell proliferation assay

EdU (Beyotime) was diluted to the recommended concentration according to the instructions to generate EdU working solution. The original cell culture medium was removed, the appropriate volume of EdU working solution was added, and the cells were incubated at 37 °C for 2 h. After EdU labeling was complete, the cells were fixed with 4% paraformaldehyde for 15 min at room temperature and washed 3 times. The cells were then incubated with 3% Triton X-100 for 10 min at room temperature and washed 3 times. Click Reaction Solution was prepared according to the manufacturer's recommendation and added to each well, and the cells were incubated for 30 min at room temperature and washed 3 times. The nuclei were stained with Hoechst 33,342 for 10 min at room temperature, and the cells were washed 3 times. After the cells were sealed with anti-fluorescence quenching agent, they were photographed with a 90I inverted fluorescence microscope.

#### Cellular reactive oxygen species (ROS) assay

DCFH-DA dye (10 mM; Beyotime) was diluted with serum-free medium at a ratio of 1:1,000. The original culture medium was removed, the appropriate volume of diluted DCFH-DA was added, and the cells were incubated for 20 min at 37 °C and then washed 3 times with serum-free medium. Images were captured using a Cytation 5.

### TUNEL staining

Apoptotic cells were detected with a TUNEL apoptosis kit (Keygen, China). Cells were fixed with 4% paraformaldehyde for 20 min and then washed with PBS 3 times. Then, 0.5% Triton X-100 was added and incubated for 10 min to induce permeabilization. The samples were subsequently stained according to the manufacturer's instructions. After mounting with an anti-fluorescence quencher, a 90I inverted fluorescence microscope was used for imaging.

### Calcium imaging

Fluo-4 AM (2 mM; Beyotime) was diluted to 3 μM using D-PBS to generate a working solution. The cells were washed three times with D-PBS, and 1 mL of Fluo-4 AM working solution was added to each dish. The cells were incubated for 40 min at 37 °C and washed three times with D-PBS. Finally, 1 mL of D-PBS was added to each dish, and the cells were incubated at 37 °C for 20 min for de-esterification. Images were captured using an A1R confocal microscope.

### Transmission electron microscopy

The samples were prefixed with 3% glutaraldehyde, postfixed with 1% osmium tetroxide, dehydrated in acetone, infiltrated with Epox 812 for a longer period of time, and embedded. The semithin sections were stained with methylene blue, and ultrathin sections were cut with a diamond knife and stained with uranyl acetate and lead citrate. Sections were examined with a JEM-1400-FLASH transmission electron microscope.

### Western blotting

Cells were lysed using lysis buffer and centrifuged at 12,000 r/min for 15 min at 4 °C, and the supernatant was subjected to SDS-PAGE at a ratio of 4:1. The protein concentration was measured using a BCA assay kit (KeyGen Biotech, China). Protein specimens were separated by electrophoresis on 10% SDS-polyacrylamide gels, and then the proteins were electrotransferred to polyvinylidenefluoride (PVDF) membranes (Millipore, USA). The membranes were blocked with TBST containing 5% milk for 1 h and then washed 3 times with TBST. The PVDF membranes were incubated with diluted primary antibodies (Table S2) overnight and then washed with TBST. The PVDF membranes was incubated with diluted secondary antibody (Table S2) for 1 h at room temperature, washed with TBST and exposed using chemiluminescence reagent (Millipore).

### RNA extraction and q-PCR

Total RNA was extracted by using RNAiso Reagent (TaKaRa, Japan) and subsequently reverse transcribed into cDNA using Takara Reverse Reagent according to the manufacturer's instructions. The forward and reverse primers for q-PCR are shown in Table S3. The cDNA templates were subsequently amplified using TB Green (TaKaRa, Japan) and the Q3 real-time quantification system. Semiquantitative analyses were performed using the ΔΔCt method.

### Mitochondrial DNA (mtDNA) copy number assay

Whole-cell DNA was extracted by using a DNA extraction kit (BioFlux, China). The level of MT-ND1 was subsequently determined by q-PCR. Semiquantitative analyses were performed using the ΔΔCt method.

### Statistical analysis

All experiments were repeated three times. When the data were normally distributed, we used unpaired Student's *t*-test or one-way analysis of variance (ANOVA). When the data were not normally distributed, we used the Mann–Whitney test or the Kruskal–Wallis test.

## Results

### Culture and identification of hiPSCs and hiPSC-CMs

**Figure 1:**
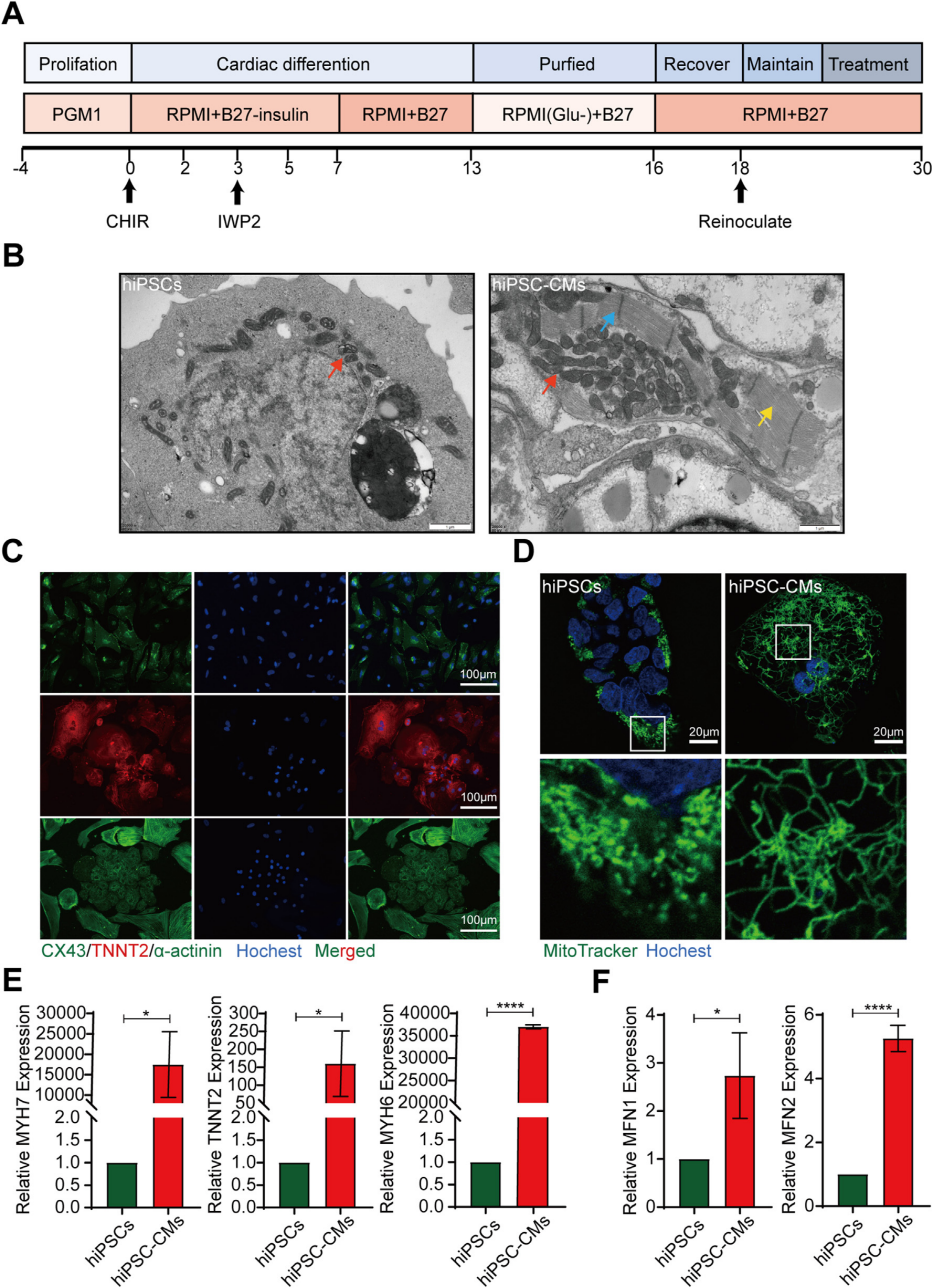
Culture and identification of human induced pluripotent stem cells (hiPSCs) and hiPSC-derived cardiomyocytes (hiPSC-CMs). **(A)** Flow chart of the protocol used to induce the differentiation of hiPSCs into hiPSC-CMs. **(B)** Electron micrographs of hiPSCs and hiPSC-CMs. Scale bar = 1 μm. Mitochondria, red arrows. Z-band, blue arrow. Myofibrils, yellow arrows. **(C)** Staining of the myocardial markers CX43, TNNT2, and α-actinin in hiPSC-CMs. Scale bar = 100 μm. **(D)** MitoTracker staining of hiPSCs and hiPSC-CMs. Scale bar = 20 μm. **(E)** q-PCR analysis of myocardial genes expression in hiPSCs and hiPSC-CMs. **(F)** Expression of *MFN1* and *MFN2* mRNAs in hiPSCs and hiPSC-CMs. The means ± SEMs are shown. ∗*P* < 0.05, ∗∗∗∗*P* < 0.0001.

**Fig. S1:**
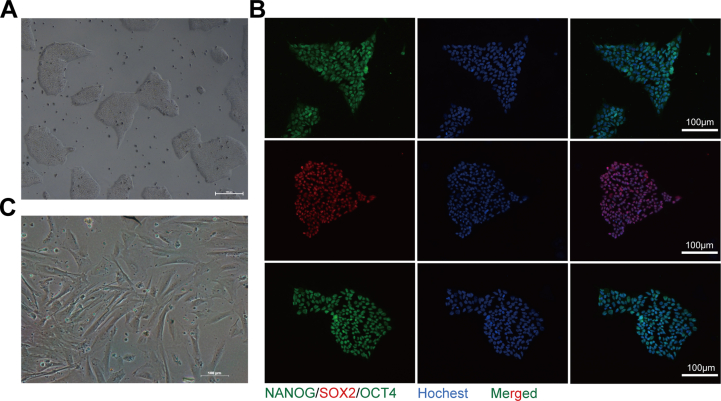
Culture and identification of hiPSCs and hiPSC-CMs. **(A)** Morphology of hiPSCs. Scale bar = 100 μm. **(B)** Immunofluorescence staining for the hiPSC stemness markers NANOG (green), SOX2 (red), and OCT4 (green). Scale bar = 100 μm. **(C)** Morphology of hiPSC-CMs on day 30. Scale bar = 100 μm.

The small-molecule inhibitors CHIR (GSK-3 inhibitor) and IWP2 (Wnt inhibitor) were used to temporally modulate Wnt signaling to induce the differentiation of hiPSCs into hiPSC-CMs,^22^ and the differentiation process is shown in Figure 1A. hiPSCs grew in clumps, had dense middles and smooth edges, were small and had a relatively large nucleoplasm (Fig. S1A). Immunofluorescence staining showed that hiPSCs expressed the stemness markers Nanog, SOX2, and OCT4 (Fig. S1B). Autonomously pulsating clumps of cells were observed on differentiation day 7 (Movie S1). After reinoculation on day 18, hiPSC-CMs appeared as monolayers with spindle-shaped morphology, the cell volume increased and the nucleoplasmic ratio decreased (Fig. S1C). The electron microscopy images showed the existence of ordered myofibrils and Z-bands in hiPSC-CMs (Fig. 1B). The immunofluorescence results showed that hiPSC-CMs expressed the cardiac markers connexin 43 (CX43), troponin T2 (TNNT2) and α-actinin (Fig. 1C). The q-PCR results also showed significantly higher expression of the myocardial-specific markers TNNT2, myosin heavy chain 6 (MYH6) and myosin heavy chain 7 (MYH7) in hiPSC-CMs than in hiPSCs (Fig. 1E). In addition, mitochondrial structure was significantly altered after differentiation. MitoTracker staining showed that the mitochondria in hiPSCs were short rod-like granules and closely surrounded the nucleus (Fig. 1D). In contrast, mitochondria in hiPSC-CMs formed networks and had elongated mitochondrial branched chains that were evenly distributed in the cytoplasm (Fig. 1D). Moreover, the electron microscopy results showed that compared with hiPSCs, mitochondria of hiPSC-CMs had more developed mitochondrial cristae (Fig. 1B). According to the q-PCR results, hiPSC-CMs exhibited increased mitochondrial fusion gene mitofusin1 (MFN1) and mitofusin2 (MFN2) expression compared with hiPSCs (Fig. 1F). These results suggest that we successfully induced the differentiation of hiPSCs into functional hiPSC-CMs.

### PINK1 deficiency causes mitochondrial fission in hiPSC-CMs

**Figure 2:**
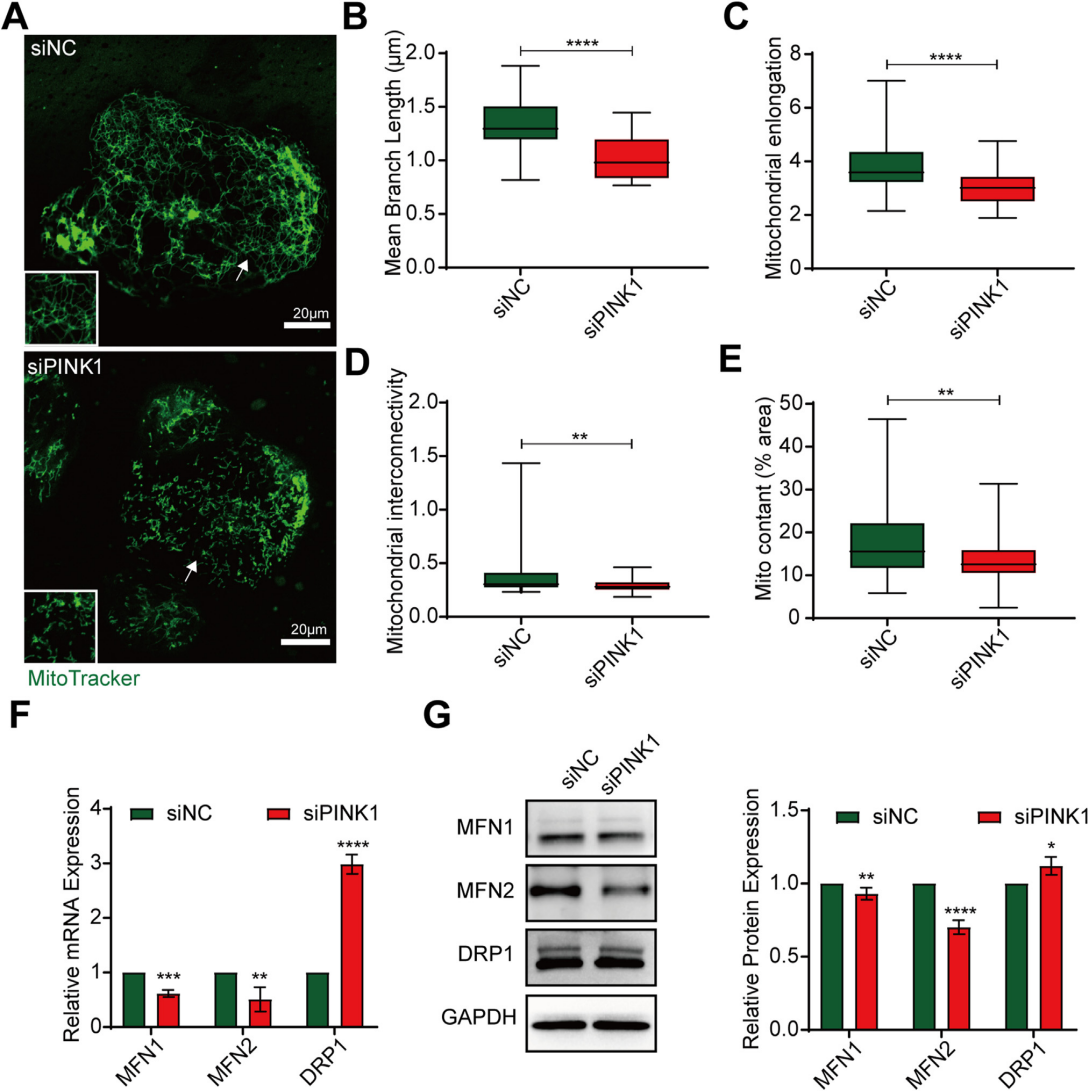
PINK1 deficiency causes mitochondrial fission in human induced pluripotent stem cell-derived cardiomyocytes (hiPSC-CMs). **(A)** MitoTracker staining of siNC- and siPINK1-transfected hiPSC-CMs. **(B**–**E)** Statistical parameters of mitochondrial morphology were determined using ImageJ software. Mean branch length (B), mitochondrial elongation (C), mitochondrial interconnectivity (D), and mitochondrial content (E). A total of 20–30 cells were analyzed in each experiment, and each experiment was repeated three times. **(F)** q-PCR analysis of *MFN1*, *MFN2*, and *DRP1* expression in siNC- and siPINK1-transfected hiPSC-CMs. **(G)** Western blotting analysis of MFN1, MFN2, and DRP1 expression in siNC- and siPINK1-transfected hiPSC-CMs. The means ± SEMs are shown. ∗∗*P* < 0.01, ∗∗∗*P* < 0.001, ∗∗∗∗*P* < 0.0001.

**Fig. S2:**
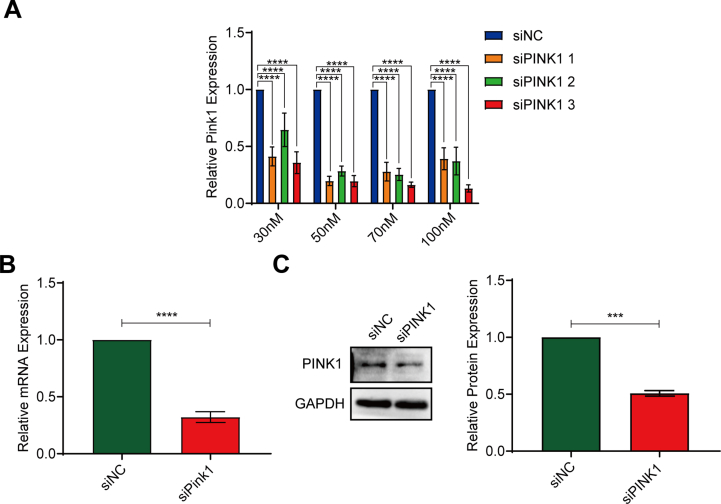
siPINK1-3 (100 nM) had the highest knockdown efficiency. **(A)** q-PCR analysis of PINK1 expression in hiPSC-CMs after treatment with 30 nM, 50 nM, 70 nM, or 100 nM siPINK1-1, siPINK1-2, or siPINK1-3. **(B)** q-PCR analysis of PINK1 expression in hiPSC-CMs after treatment with 100 nM siPINK1 for 48 h. **(C)** Western blotting analysis of PINK1 expression in hiPSC-CMs after treatment with 100 nM siPINK1 for 48 h.

To investigate the effect of PINK1 on mitochondrial structure in hiPSC-CMs, we used siRNA to knock down PINK1 expression in hiPSC-CMs. First, we identified the siRNA sequences with the highest knockdown efficiency. The q-PCR results showed that the third siPINK1 sequence had the highest knockdown efficiency at a concentration of 100 nM (Fig. S2A). We then validated this finding using q-PCR and Western blotting, which showed that intracellular PINK1 expression was significantly suppressed after siPINK1 transfection (Fig. S2B, C). Mitochondria are divided into punctate, rod-like (monomeric) and branching structures (reticular) according to their morphology,^20^ and mitochondria are generally distributed in a network-like pattern in mammalian cells. We then used MitoTracker to stain mitochondria. Our results showed that siPINK1 caused the mitochondria of hiPSC-CMs to fragment from the fused reticulum into short rods (Fig. 2A–D), and the mitochondrial content decreased (Fig. 2E). We then examined the expression of mitochondrial fusion and fission genes. The mRNA and protein levels of the mitochondrial fusion genes MFN1 and MFN2 were significantly reduced after siPINK1 transfection, while the mRNA and protein levels of the mitochondrial fission gene dynamin-related protein 1 (DRP1) were increased (Fig. 2F, G). These results suggest that inhibiting PINK1 expression in hiPSC-CMs destabilizes the mitochondrial structure and lead to mitochondrial fragmentation.

### PINK1 deficiency inhibits mitophagy in hiPSC-CMs

**Figure 3:**
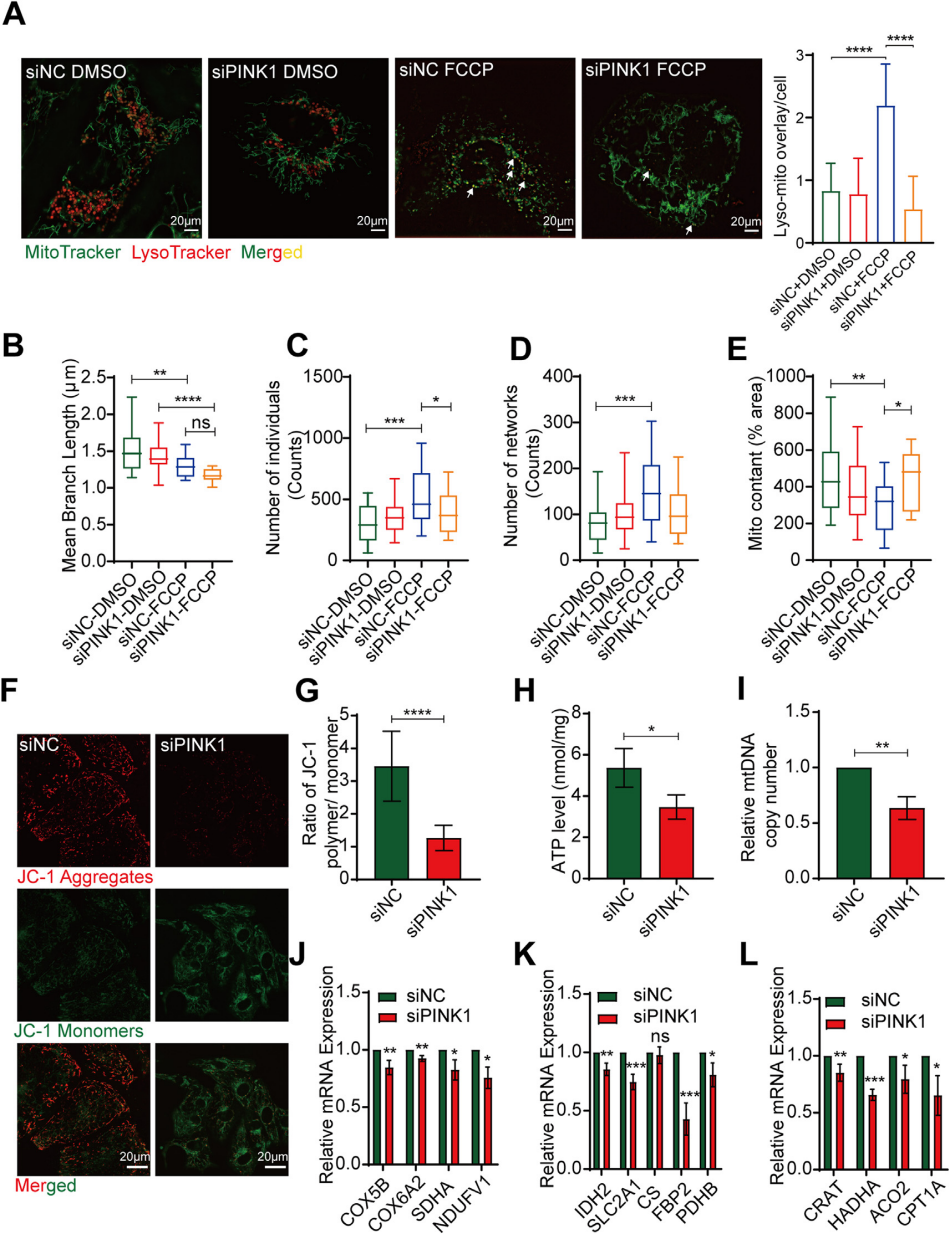
PINK1 deficiency inhibits mitophagy and mitochondrial function in human induced pluripotent stem cell-derived cardiomyocytes (hiPSC-CMs). **(A)** Staining of mitochondria (MitoTracker) and lysosomes (LysoTracker) in siNC- and siPINK1-transfected hiPSC-CMs. ImageJ software was used to determine the colocalization area of mitochondria and lysosomes. A total of 20–30 cells were analyzed in each experiment, and each experiment was repeated three times. **(B**–**E)** Statistical parameters of mitochondrial morphology were determined using ImageJ software. Mean branch length (B), number of mitochondrial individuals (C), number of mitochondrial networks (D), and mitochondrial content (E). **(F, G)** The ΔΨm of siNC- and siPINK1-transfected hiPSC-CMs. JC-1 aggregates (red) and JC-1 monomers (green) are shown. Scale bar = 20 μm. **(H)** ATP content in siNC- and siPINK1-transfected hiPSC-CMs. **(I)** mtDNA copy number in siNC- and siPINK1-transfected hiPSC-CMs. **(J**–**L)** The mRNA expression of mitochondrial electron respiration transport chain-related genes (J), glycolysis and tricarboxylic acid cycle genes (K), and fatty acid oxidation-related genes (L) in siNC- and siPINK1-transfected hiPSC-CMs. The means ± SEMs are shown. ∗*P* < 0.05, ∗∗*P* < 0.01, ∗∗∗*P* < 0.001, ∗∗∗∗*P* < 0.0001.

Mitophagy is an important biological process by which damaged mitochondria are cleared from the cell, preventing the accumulation of damaged mitochondria, which can cause cellular damage.^23^ When the mitochondrial membrane potential (ΔΨm) is reduced, PINK1 is stabilized on the outer mitochondrial membrane,^24^ where it recruits and phosphorylates the downstream ubiquitin ligase Parkin to ubiquitinate the substrate,^9^ followed by lysosomal recognition and phagocytosis of the ubiquitin-labeled mitochondria. We treated hiPSC-CMs with siPINK1 and used the respiratory chain uncoupling agent FCCP to activate PINK1-mediated mitophagy by decreasing the ΔΨm for a short period of time.^25^ MitoTracker-LysoTracker staining showed that the siNC group showed increased mitophagy and a larger yellow colocalization area in the cells, while the siPINK1 group showed diminished mitophagy (Fig. 3A). Additionally, the length of the mitochondrial branch was significantly reduced in the siNC- and siPINK1-FCCP groups compared to the siNC- and siPINK1-DMSO groups (Fig. 3B). The numbers of mitochondrial individuals and mitochondrial networks were significantly increased in the siNC-FCCP group compared to the siNC-DMSO group (Fig. 3C, D). In contrast, the number of individuals in the siPINK1-FCCP group was reduced compared to that in the siPINK1-DMSO group (Fig. 3C). Furthermore, the mitochondrial content of the siNC group was significantly reduced after the treatment with FCCP (Fig. 3E). Compared with the siNC-FCCP group, mitochondria in the siPINK1-FCCP group appeared to accumulate (Fig. 3E). Taken together, these results suggest that siPINK1 inhibits mitophagy in hiPSC-CMs.

### PINK1 deficiency inhibits the biological function of mitochondria in hiPSC-CMs

**Fig. S3:**
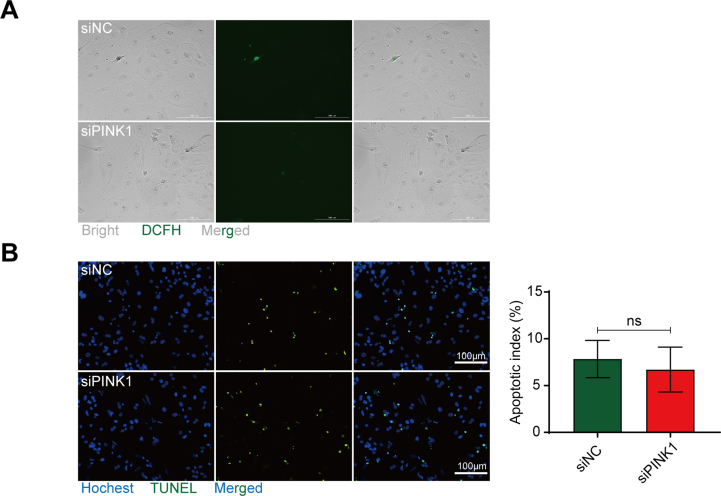
Inhibition of PINK1 expression does not damage hiPSC-CMs. **(A)** DCF staining of siNC- and siPINK1-transfected hiPSC-CMs. DCF staining (green). Scale bar = 200 μm. **(B)** TUNEL staining of siNC- and siPINK1-transfected hiPSC-CMs. Hoechst 33342 (blue) and TUNEL (green) staining are shown. Scale bar = 100 μm.

Because of the inextricable link between mitochondrial structure and function,^26^ we hypothesized that mitochondrial fragmentation caused by siPINK1 affects normal mitochondrial functions. Maintaining a stable ΔΨm is an important prerequisite for continuous mitochondrial ATP production.^27^ We investigated the effect of PINK1 on the ΔΨm of hiPSC-CMs by staining the mitochondria with JC-1. The ΔΨm of siPINK1- transfected hiPSC-CMs was significantly reduced compared with that of siNC- transfected hiPSC-CMs (Fig. 3F, G). ATP production depends on the potential gradient formed by the inner mitochondrial membrane.^28^ Accompanied by a decrease in ΔΨm, ATP levels were significantly reduced in siPINK1- transfected hiPSC-CMs (Fig. 3H). The expression of mitochondrial respiratory chain complexes, as well as energy metabolism-related metabolic enzymes, was significantly reduced in siPINK1- transfected hiPSC-CMs compared to siNC- transfected hiPSC-CMs (Fig. 3J–L). We also assessed the mtDNA copy number to evaluate mitochondrial biogenesis and found that the mtDNA copy number was reduced after siPINK1 transfection (Fig. 3I). Surprisingly, siPINK1 did not cause a significant increase in intracellular ROS production (Fig. S3A) or apoptosis (Fig. S3B). Taken together, siPINK1 inhibits mitochondria-related biological functions independent of ROS accumulation and apoptosis.

### PINK1 deficiency suppresses hiPSC-CMs maturation

**Figure 4:**
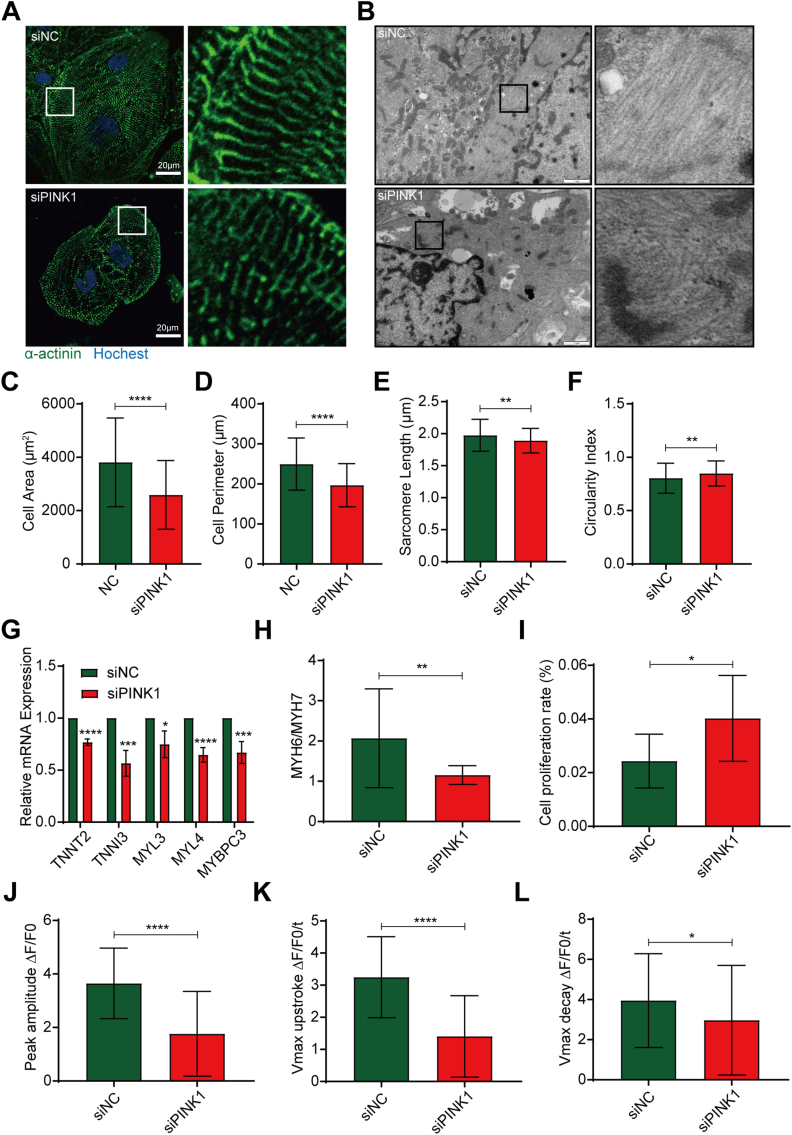
PINK1 deficiency suppresses human induced pluripotent stem cell-derived cardiomyocytes (hiPSC-CMs) maturation. **(A)** Hoechst 33,342 (blue) staining and α-actinin (green) immunofluorescence in siNC- and siPINK1-transfected hiPSC-CMs. Scale bar = 20 μm. **(B)** Electron micrographs of siNC- and siPINK1-transfected hiPSC-CMs. Scale bar = 1 μm. **(C–F)** The morphological indicators of hiPSC-CMs determined with ImageJ software. Cell area **(**C), cell perimeter (D), sarcomere length (E) and circularity index (F). **(G)** q-PCR analysis of cardiac marker expression in siNC- and siPINK1-transfected hiPSC-CMs. **(H)** Ratio of MYH6 to MYH7. **(I)** Proliferation rates of siNC- and siPINK1-transfected hiPSC-CMs. **(J**–**L)** Calcium transients in siNC- and siPINK1-transfected hiPSC-CMs. Maximum calcium transient amplitude (J), calcium transient maximum rise rate (K), and calcium transient maximum fall rate (L). The means ± SEMs are shown. ∗*P* < 0.05, ∗∗*P* < 0.01, ∗∗∗*P* < 0.001, ∗∗∗∗*P* < 0.0001.

We next investigated the effect of PINK1 on the maturation of hiPSC-CMs. The maturation of hiPSC-CMs was accompanied by a gradual increase in the cell area, perimeter, sarcomeres, and a decrease in the circularity index.^29^ α-actinin was stained in PINK1 knockdown hiPSC-CMs(Fig. 4A). The cell area (Fig. 4C), cell perimeter (Fig. 4D), and sarcomere length (Fig. 4E) were significantly decreased in siPINK1-transfected hiPSC-CMs compared with siNC-transfected hiPSC-CMs. The circularity index was increased in siPINK1-transfected hiPSC-CMs compared with siNC-transfected hiPSC-CMs (Fig. 4F). These results suggest that the morphology of hiPSC-CMs tended to be more roundness. Moreover, the electron microscopy results showed that myofibrils in the siPINK1 group were disordered compared with the siNC group (Fig. 4B).

The myocardial contraction force depends on the integrity of myocardial structural units and the strength of the Ca^2+^ handling capacity.^30^^,^^31^ To clarify the effect of siPINK1 on myocardial contractile function, we first examined the expression of myocardial structure-related genes. The expression of myosin and troponin was significantly reduced (Fig. 4G). A shift in myosin heavy chain isoforms and a shift from MYH6 to MYH7 occurs during CM maturation.^32^ Our results showed that the MYH6/MYH7 ratio was decreased (Fig. 4H). We then examined the calcium transient kinetics. Compared with siNC-transfected hiPSC-CMs, siPINK1-transfected hiPSC-CMs showed a reduction in the peak calcium transient amplitude (Fig. 4J), and significant reductions in the maximum velocity of upstroke and the maximum velocity of decay (Fig. 4K, L).

**Fig. S4:**
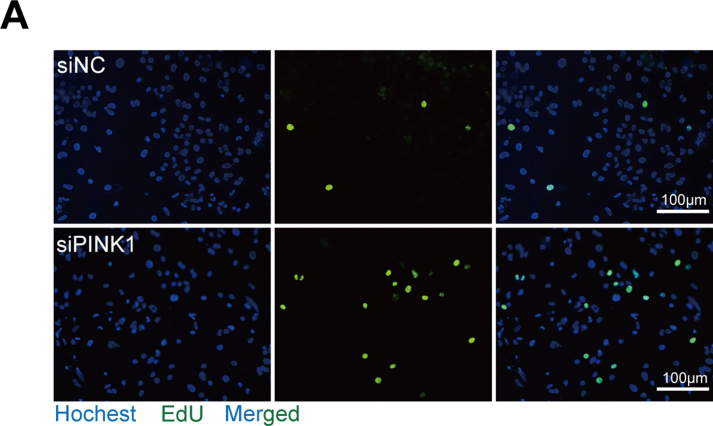
Inhibition of PINK1 expression increases the proliferation of hiPSC-CMs. Hoechst (blue) and EdU (green) staining are shown. Scale bar = 100 μm.

As CMs mature, they gradually lose their proliferative capacity and undergo hypertrophy.^33^ We used EdU staining to examine the effect of siPINK1 on the proliferation of hiPSC-CMs. Compared with siNC-transfected hiPSC-CMs, the proliferation rate of siPINK1-transfected hiPSC-CMs increased (Fig. 4I; Fig. S4). These results suggest that PINK1 deficiency inhibits the maturation of hiPSC-CMs.

### An increase in mitochondrial fusion rescues siPINK1-induced mitochondrial structural abnormalities and partial dysfunction

**Figure 5:**
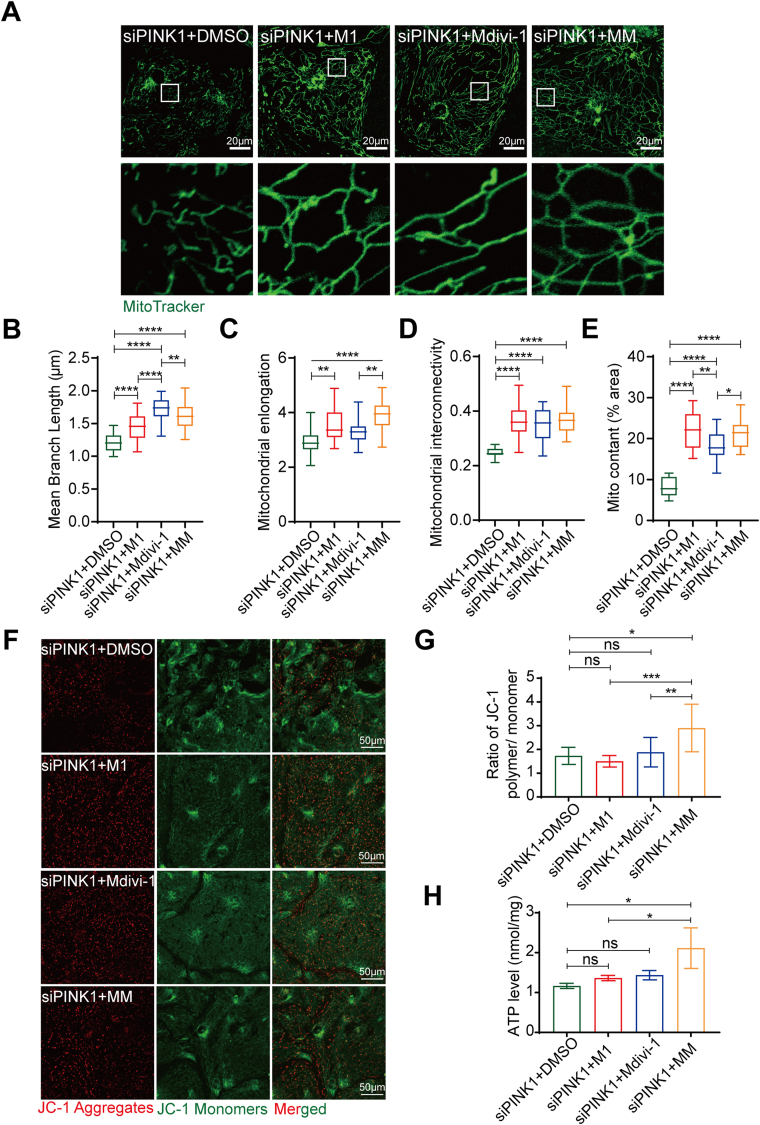
The induction of mitochondrial fusion rescues siPINK1-induced mitochondrial structural abnormalities and partial dysfunction. **(A)** MitoTracker staining of siPINK1-transfected human induced pluripotent stem cell-derived cardiomyocytes (hiPSC-CMs) treated with DMSO, M1, Mdivi-1 and Mdivi-1+M1 (MM). Scale bar = 20 μm. **(B**–**E)** Statistical analysis of mitochondrial morphological parameters using ImageJ software. Average branch length (B), mitochondrial elongation (C), mitochondrial interconnectivity (D), and mitochondrial content (E). **(F)** ΔΨm of siPINK1-transfected hiPSC-CMs treated with DMSO, M1, Mdivi-1 and Mdivi-1+M1 (MM). **(G)** The ratio of red to green fluorescence indicates the ΔΨm. **(H)** Intracellular ATP contents of siPINK1-transfected hiPSC-CMs treated with DMSO, M1, Mdivi-1 and Mdivi-1+M1 (MM). The means ± SEMs are shown. ∗*P* < 0.05, ∗∗*P* < 0.01, ∗∗∗∗*P* < 0.0001.

**Fig. S5:**
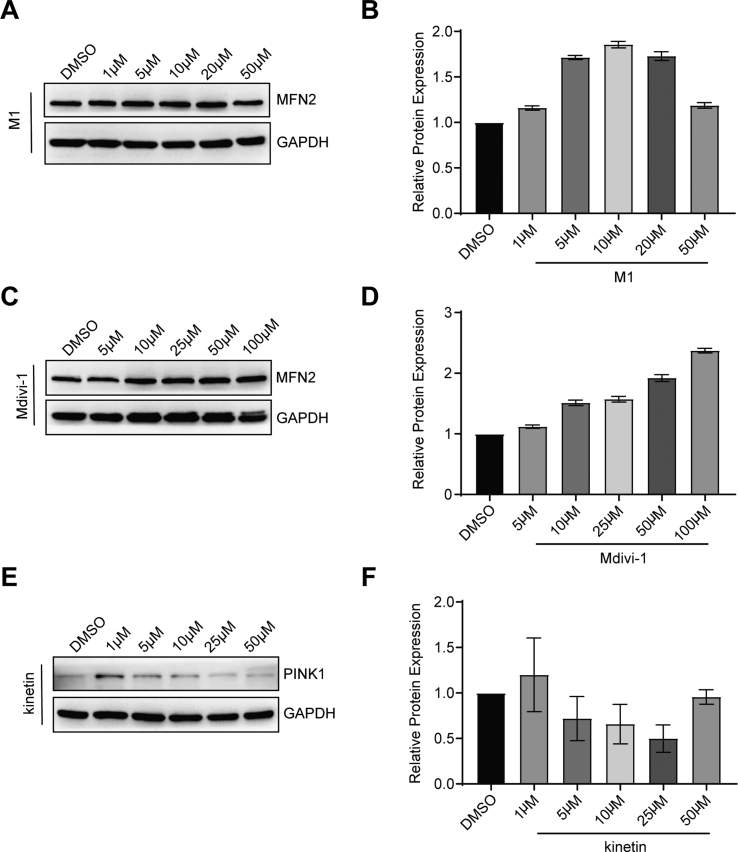
The optimal concentrations of M1, Mdivi-1 and kinetin were determined. **(A)** Western blotting analysis of MFN2 expression in hiPSC-CMs after treatment with different concentrations of M1. **(B)** Statistical graph of the results shown in **(A)**. **(C)** Western blotting analysis of MFN2 expression in hiPSC-CMs after treatment with different concentrations of Mdivi-1. **(D)** Statistical graph of the results shown in **(C)**. **(E)** Western blotting analysis of PINK1 expression in hiPSC-CMs after treatment with different concentrations of kinetin. **(F)** Statistical graph of the results shown in **(E)**.

To further clarify whether PINK1 affects mitochondrial function by maintaining mitochondrial structural homeostasis in hiPSC-CMs, we treated siPINK1-transfected hiPSC-CMs with the mitochondrial fusion promoter M1^34^ and the mitochondrial fission inhibitor Mdivi-1.^12^ We first determined the optimal concentrations of M1 and Mdivi-1. The Western blotting results showed that the most pronounced increase in MFN2 expression was observed in response to 10 μM M1 (Fig. S5A, B) and 100 μM Mdivi-1 (Fig. S5C, D). MitoTracker staining showed that treatment with M1 and Mdivi-1 rescued mitochondrial fragmentation in siPINK1-transfected hiPSC-CMs (Fig. 5A), and significantly increased the mitochondrial branch length (Fig. 5B), mitochondrial elongation (Fig. 5C) and mitochondrial interconnectivity (Fig. 5D). Additionally, the mitochondrial content was significantly increased (Fig. 5E). Thus, promoting mitochondrial fusion or inhibiting mitochondrial fission significantly ameliorates mitochondrial fragmentation in siPINK1-transfected hiPSC-CMs.

We then examined whether a change in the mitochondrial structure reverses the mitochondrial dysfunction caused by siPINK1. Next, we measured the ΔΨm of siPINK1-transfected hiPSC-CMs after M1 and Mdivi-1 treatment (Fig. 5F). The results showed no significant increases in the ΔΨm of siPINK1-transfected hiPSC-CMs after M1 or Mdivi-1 treatment (Fig. 5F, G). We subsequently examined the ATP levels, and the results showed that there was no significant change in cells treated with M1 or Mdivi-1 alone (Fig. 5H). In summary, the mitochondrial structural abnormalities and dysfunction caused by PINK1 deletion may be independent processes, and rescuing mitochondrial fragmentation does not effectively improve mitochondrial function.

### PINK1 kinase activity enhances mitochondrial and morphological maturation of hiPSC-CMs

**Figure 6:**
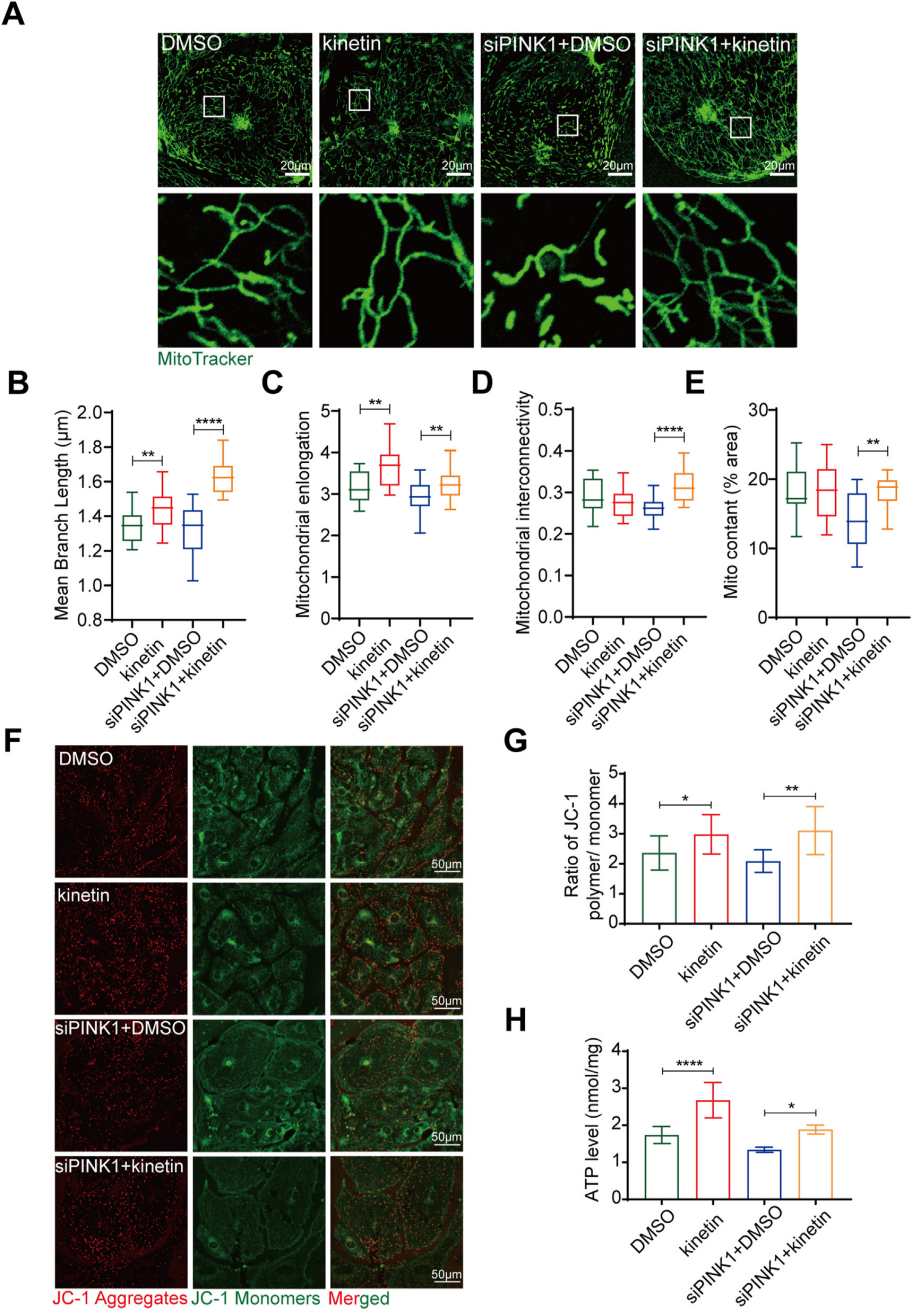
PINK1 kinase activity enhances mitochondrial maturation of human induced pluripotent stem cell-derived cardiomyocytes (hiPSC-CMs). **(A)** MitoTracker staining of DMSO-, kinetin-treated hiPSC-CMs and siPINK1-transfected hiPSC-CMs treated with DMSO and kinetin. Scale bar = 20 μm. **(B**–**E)** Statistical analysis of mitochondrial morphological parameters using ImageJ software. Average branch length (B), mitochondrial elongation (C), mitochondrial interconnectivity (D), and mitochondrial content (E). **(F)** ΔΨm of DMSO-, kinetin-treated hiPSC-CMs and siPINK1-transfected hiPSC-CMs treated with DMSO and kinetin. **(G)** The ratio of red to green fluorescence indicates the ΔΨm. **(H)** Intracellular ATP contents of DMSO-, kinetin-treated hiPSC-CMs and siPINK1-transfected hiPSC-CMs treated with DMSO and kinetin. The means ± SEMs are shown. ∗∗*P* < 0.01, ∗∗∗∗*P* < 0.0001.

**Fig. 7:**
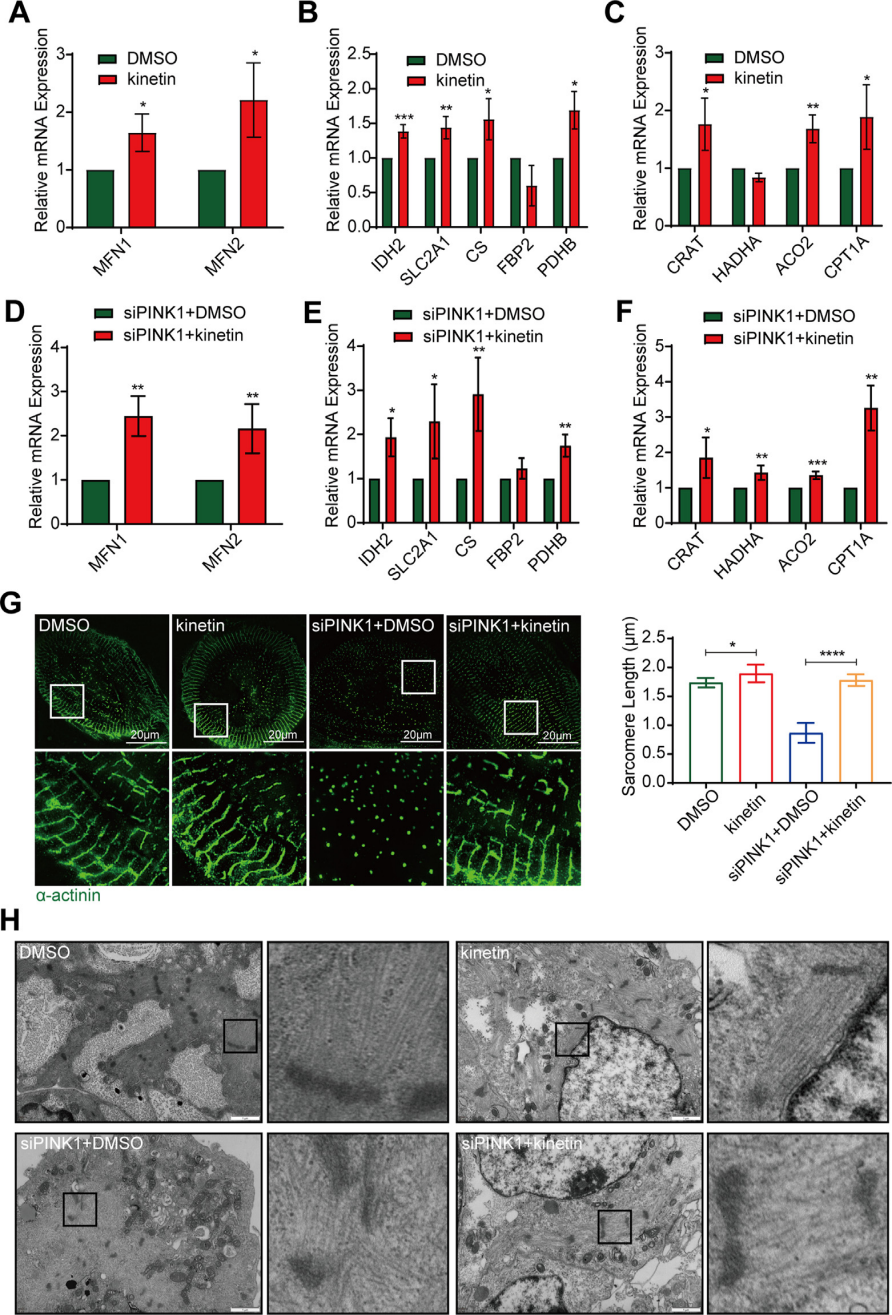
Activation of PINK1 affects the maturation of human induced pluripotent stem cell-derived cardiomyocytes (hiPSC-CMs). **(A**–**C)** The mRNA expression of genes related to mitochondrial fusion (A), glycolysis, the tricarboxylic acid cycle (B), and fatty acid oxidative metabolism (C) in DMSO-, kinetin-treated hiPSC-CMs. **(D**–**F)** The mRNA expression of genes related to mitochondrial fusion (D), glycolysis, the tricarboxylic acid cycle (E), and fatty acid oxidative metabolism (F) in siPINK1-transfected hiPSC-CMs treated with DMSO and kinetin. **(G)** α-Actinin (green) immunofluorescence staining and Hoechst 33,342 (blue) staining in DMSO-, kinetin-treated hiPSC-CMs and siPINK1-transfected hiPSC-CMs treated with DMSO and kinetin. Scale bar = 20 μm. ImageJ software was used to determine the sarcomere length in hiPSC-CMs. **(H)** Electron micrographs of DMSO-, kinetin-treated hiPSC-CMs and siPINK1-transfected hiPSC-CMs treated with DMSO and kinetin. Scale bar = 1 μm. The means ± SEMs are shown. ∗*P* < 0.05, ∗∗*P* < 0.01, ∗∗∗*P* < 0.001, ∗∗∗∗*P* < 0.0001.

PINK1 functions as a serine/threonine kinase by phosphorylating downstream proteins.^18^^,^^35^ Studies have shown that the absence of PINK1 activity in the heart reduces cardiac mitochondrial function.^17^ Kinetin is an ATP analog that is taken up by cells, processed intracellularly and converted to KTP, a novel activator of PINK1 kinase activity.^36^ The Western blotting results showed that 1 μM kinetin significantly activated PINK1 (Fig. S5E, F). MitoTracker staining (Fig. 6A) showed that the mitochondrial branch length (Fig. 6B) and mitochondrial elongation (Fig. 6C) increased after the activation of PINK1 kinase activity in hiPSC-CMs, but mitochondrial interconnectivity (Fig. 6D) and mitochondrial content (Fig. 6E) were not significantly changed. However, treatment of siPINK1-transfected hiPSC-CMs with kinetin significantly rescued mitochondrial fragmentation (Fig. 6A) and increased the mitochondrial branch length (Fig. 6B), mitochondrial elongation (Fig. 6C), mitochondrial interconnectivity (Fig. 6D) and mitochondrial content (Fig. 6E). The q-PCR results were consistent with the results of MitoTracker staining (Fig. 7A, D). These results suggest that PINK1 kinase activity is necessary for the stabilization of mitochondrial structure.

The results described above suggest that rescuing the mitochondrial structure alone does not reverse the mitochondrial dysfunction caused by PINK1 deletion, and we therefore hypothesized that PINK1 may regulate mitochondrial function through its kinase activity. We then measured the ΔΨm of siPINK1-transfected hiPSC-CMs after kinetin treatment. The activation of PINK1 in hiPSC-CMs increased the ΔΨm (Fig. 6F, G) and intracellular ATP levels (Fig. 6H). Activating PINK1 in siPINK1-transfected hiPSC-CMs reversed the decrease in ΔΨm (Fig. 6F, G) and increased intracellular ATP levels (Fig. 6H). The mRNA expression of glycolysis-, tricarboxylic acid cycle- and fatty acid oxidation-related genes were significantly upregulated by kinetin in hiPSC-CMs (Fig. 7B, C). Also, treatment of siPINK1-transfected hiPSC-CMs with kinetin significantly increased metabolism-related genes expression (Fig. 7E, F). These results suggest that PINK1 kinase activity is necessary to maintain the functional stability of mitochondria in hiPSC-CMs.

**Fig. S6:**
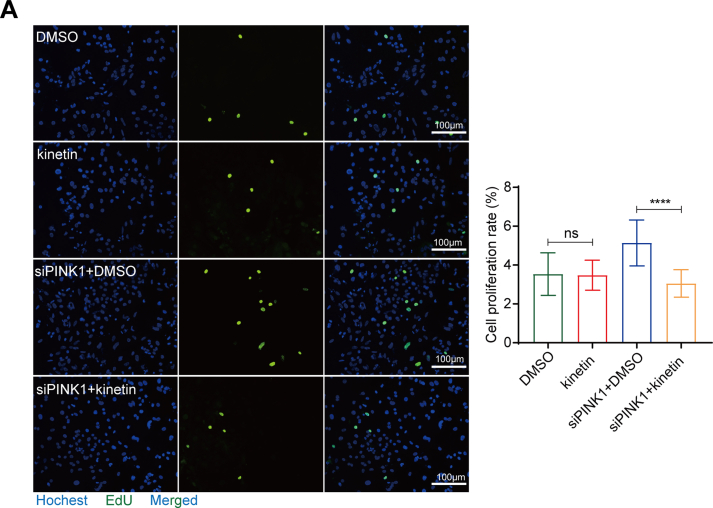
PINK1 activation reduces the proliferation of hiPSC-CMs when PINK1 expression is inhibited. Hoechst (blue) and EdU (green) staining are shown. Scale bar = 100 μm.

Furthermore, hiPSC-CMs exhibited longer and well-aligned sarcomeres after kinetin administration (Fig. 7G) and that the proliferation rate of the cells was reduced (Fig. S6). The electron microscopy results showed that the myofibrils of the kinetin-treated hiPSC-CMs were neatly arranged, which was not significantly different from that of the DMSO group (Fig. 7H). However, the myofibril arrangement was disordered in the siPINK1-transfected hiPSC-CMs treated with DMSO compared to the siPINK1-transfected hiPSC-CMs treated with kinetin (Fig. 7H). Taken together, PINK1 kinase activity is necessary to maintain structural homeostasis and the functional stability of mitochondria in hiPSC-CMs and to mediate hiPSC-CMs maturation.

## Discussion

Our results show that PINK1 plays an important role in maintaining the structure and function of the mitochondria in hiPSC-CMs under physiological conditions and facilitating their maturation. When hiPSC-CMs were transfected with siPINK1, they exhibited mitochondrial fragmentation, a reduced ΔΨm, and a decreased cellular energy supply. And the structural and functional maturation of hiPSC-CMs were limited. Furthermore, the induction of mitochondrial fusion rescued the mitochondrial fragmentation caused by PINK1 deletion and partially rescued the mitochondrial function. Activation of PINK1 kinase activity significantly rescued the structure and function of fragmented mitochondria and mediated hiPSC-CMs maturation. Thus, our results demonstrate that PINK1 is essential for maintaining mitochondrial quality in hiPSC-CMs under physiological conditions and for promoting the maturation of hiPSC-CMs.

PINK1 expression is important for the maintenance of normal mitochondrial quality and biological function in hiPSC-CMs. Numerous studies have shown that PINK1 deficiency leads to mitochondrial fragmentation.^13^^,^^37^^,^^38^ In our study, inhibition of PINK1 expression in hiPSC-CMs destabilized the mitochondrial structure and caused the fragmentation of mitochondria from fused networks to punctate or rod-shaped structures. In addition, we observed elevated DRP1 expression following siPINK1 transfection. Mitochondrial fission is primarily driven by DRP1, which oligomerizes to drive mitochondrial fragmentation upon binding to the corresponding receptor on the mitochondrial outer membrane.^26^ Single or combined use of M1 and Mdivi-1 ameliorated the mitochondrial fragmentation caused by PINK1 deletion. However, rescuing the mitochondrial structure alone did not increase the ΔΨm or ATP production, possibly because mitochondrial organization and function are regulated by PINK1 through different mechanisms. PINK1 deletion causes the absence of mitochondrial respiratory chain complexes I and III,^14^ reducing the production of ATP. Additionally, PINK1 deletion impairs the mitochondrial uptake of Ca^2+^,^13^ which also leads to a reduction in ATP synthesis. Consistently with our results, PINK1 deletion reduces mitochondrial respiratory chain gene expression, but further studies are needed to verify whether these changes are associated with impaired mitochondrial Ca^2+^ uptake. Surprisingly, PINK1 deletion did not lead to an increase in intracellular ROS production in the present study. Excessive ROS production leads to mitochondrial fragmentation^39^ and a decrease in ΔΨm, thereby reducing mitochondrial bioenergetic synthesis. It is possible that PINK1 directly regulates the mitochondrial structure in hiPSC-CMs and that the observed fragmentation is not due to mitochondrial damage. Taken together, these findings suggest that PINK1 mediates the structural and functional stabilization of mitochondria in hiPSC-CMs.

Although numerous studies have clearly shown that the deletion of PINK1 in the adult heart disrupts of mitochondrial structural stability and results in a loss of function, the mechanism by which PINK1 maintains mitochondrial quality during cardiac development has rarely been explored.^17^^,^^40^^,^^41^ PINK1 kinase activity is mainly mediated by its C-terminal functional domain,^10^^,^^42^ and studies have shown that kinetin restores PINK1 kinase activity in mutant cells.^36^ According to our results, under normal physiological conditions, treatment with kinetin does not significantly alter the mitochondrial structure, whereas it rescues the mitochondrial structure and function in cells with lower expression of PINK1. This suggests that PINK1 may regulate the mitochondrial structure and function of hiPSC-CMs through its kinase activity. Therefore, the identification of the potential mitochondrial substrates on which PINK1 kinase acts is important to clarify how PINK1 affects mitochondrial structure and function.

Fetal cardiomyocytes have fewer and smaller sarcomeres, lower maximal contractility, slower ascending velocity, higher resting potential, no T-tubules, and they rely on glycolysis as the primary energy source.^33^^,^^43^ In contrast, mature adult cardiomyocytes have more organized and longer sarcomeres, exhibit lower resting membrane potential and higher T-tubule formation, and are dependent on oxidative phosphorylation for ATP production.^44^ Our results showed that PINK1 deletion led to decreased expression of myocardial structure-related genes, disordered myofibril arrangement in hiPSC-CMs, and reduced calcium transient kinetics. Treatment with kinetin, an activator of the kinase activity of PINK1, significantly improved the myofibril arrangement. Interestingly, it has also been shown that the PINK1 levels are significantly reduced in patients with end-stage heart failure.^17^ In a previous study, *PINK1* knock-out mice exhibited abnormal cardiac mitochondrial function and elevated oxidative stress, with early left ventricular dysfunction and pathological cardiac hypertrophy.^17^ Our result suggests that the loss of PINK1 may be related to developmental disorders of cardiomyocytes. Defective cardiomyocyte maturation due to lack of PINK1 may ultimately the development of lead to heart diseases.

In conclusion, PINK1 is critical for the developmental maturation of hiPSC-CMs, with apparent roles in the maintenance of mitochondrial structure and function. Further studies exploring the mechanisms by which PINK1 regulates mitochondrial quality are critical to identify methods that can promote the maturation of hiPSC-CMs.

## Author contributions

**Huiwen Liu:** conceptualization, methodology, formal analysis, investigation, writing—original draft, and writing—review & editing. **Yanting Sun:** formal analysis and writing—review & editing. **Hao Xu:** formal analysis and writing—review & editing. **Bin Tan:** formal analysis and writing—review & editing. **Qin Yi:** resources and project administration. **Jie Tian:** supervision and writing—review & editing. **Jing Zhu:** conceptualization, supervision, and writing—review & editing.

## Conflict of interests

The authors have no competing interests to declare.

## Funding

This research was supported by the National Natural Science Foundation of China (No.81970244) and General Basic Research Project from the Ministry of Education Key Laboratory of Child Development and Disorders, China (No. GBRP-202108).
