# Supplementary material for: PTEN-induced putative kinase 1 regulates mitochondrial quality control and is essential for the maturation of human induced pluripotent stem cell-derived cardiomyocytes
Source: Genes Dis. 2022 Sep 10;10(5):2151–66. doi: 10.1016/j.gendis.2022.08.023 (PMC10363588; doi:10.1016/j.gendis.2022.08.023)
Supplement: Multimedia component 2 [file mmc2.docx]

**Table 1.**

Table 1. siRNA sequences for PINK1

| Sequences name | | siRNA sequences |
| --- | --- | --- |
| GenOFFTM st-h-PINK1_001  GenOFFTM st-h-PINK1_002  GenOFFTM st-h-PINK1_003 | GGACGCTGTTCCTCGTTAT  CCAACAGGCTCACAGAGAA  GGCTGGTGATCGCAGATTT | |

**Table 2.**

Table 2. The antibodies used for Immunofluorescence and Western blot

| Antibodies | Manufacturer | Assay | Cat. No. |
| --- | --- | --- | --- |
| α-actinin | Proteintech | IF | 11313-2-AP |
| cTnT | Proteintech | IF | 15513-1-AP |
| CX43 | Proteintech | IF | 26980-1-AP |
| NANOG | Proteintech | IF | 14295-1-AP |
| SOX2 | Proteintech | IF | 11064-1-AP |
| OCT4 | Proteintech | IF | 11263-1-AP |
| FITC-conjugated goat anti-rabbit IgG | ZSGB-BIO | IF | ZF0311 |
| Cy3-conjugated goat anti-mouse IgG | Proteintech | IF | SA00009-1 |
| MFN1 | Proteintech | WB | 13798-1-AP |
| MFN2 | Proteintech | WB | 12186-1-AP |
| PINK1  GAPDH  goat anti-rabbit IgG | Proteintech  Affinity  Proteintech | WB  WB  WB | 23274-1-AP  AF7021  B900210 |

**Table 3.**

Table 3. Primer sequences for qRT-PCR

| Target Gene | Forward Primer Sequence (5’ to 3’) | Reverse Primer Sequence (5’ to 3’) |
| --- | --- | --- |
| PINK1  TNNT2  MYH6  MYH7  TNNI3  MYL3  MYL4  MYBPC3  MFN1  MFN2  DRP1  IDH2  FBP2  CRAT  HADHA  CPT1A  COX5B  COX6A2  SDHA  NDUFV1 | GCCCGAGTAGCCGCAAATGTG  TTCACCAAAGATCTGCTCCTCGCT  GTTCAAGAAGATAGTGGAACGC  GCTTCGGGAAATTCATTCGAAT  CTCCAACTACCGCGCTTATG  GCCCTAAGGAGGTCGAGTTT  GACTTCACTGCCGACCAGAT  GGCATGCTAAAGAGGCTCAA  TGAGGCAGTTTGGCATCTGT  CAATCTGAGGCGACTGGTGA  TGCTTCCCAGAGGTACTGG  GTGGAGACGGTGGAGAGTGGAG  TGAGGTGAAGAAACTGGATGTGCTATC  CGCTTCAAGGCACACCAGGATG  GGTGGATGAAGTTGGTGTGGATGTAG  GTGAGCGACTGGTGGGAGGAG  TGTGAAGAGGACAATACCAGC  TCCCTACCAACACCTCCGCATC  AAGACTACAAGGTGCGGATTGATGAG  GTGGACTGGATGAACAAGGTGATGG | GGAGCCAGCCAACCATCTTGTC  TTATTACTGGTGTGGAGTGGGTGTGG  GGCTTGATCTTGAAGTAGAGCT  TCAGGCTTTTTGTTAGACAGGA  CTCGCTCCAGCTCTTGCTTT  ACACTGCCCGTAGGTGATCT  CTCGGCATTGGTAGGGTTCT  TCTTGTGGCCTTTGCTCAC  CGCCTTCTTAGCCAGCACAA  GTCCTGACTTCACCTTCCCG  CCATGTAGCAGGGTCATTTTCT  GGTGTTCAGGAAGTGCTCGTTCAG  CTTCTCCTTGGCGGTGATGATGG  CACTCCTCCTCACTCACGATGGG  ATCTGTGTCAGCAGTTCTGGGTTTC  TGCTGCCTGAATGTGAGTTGGAAG  CCAGCTTGTAATGGGCTCC  ACGTGGCTATTGTGGAACAGAGTG  TGTGCTTCCTCCAGTGCTCCTC  GCCTTCTATCTGCTTGCTGATCTCC |
| PDHB | GAGGGCTGTGGAAGAAATATGGAGAC | AATGGCTTGCATGGAGAAATTGAAGG |
| SLC2A1 | GATGAAGGAAGAGAGTCGGCAGATG | CAGCACCACAGCGATGAGGATG |
| ACO2 | CTTGAGGGATCTGGGTGGCATTG | TGTTGTAGGAGGTGACGATTGTGTTC |
| CS | GACTTTCAGGCAGCAACATGG | ACTAAAGCCTCGGAAACGGA |
| MT-ND1 | GGCTACATACAATTACGCAAAG | TAGAATGGAGTAGACCGAAAGG |
| β-Globin | GTGCTCGGTGCCTTTAGTGA | GGGGAAAGAAAACATCAAGCGT |
| GAPDH | GAAATCCCATCACCATCTTCCAG | AAATGAGCCCCAGCCTTCTC |
